# The role of RUNX1/NF-κB in regulating PVAT inflammation in aortic dissection

**DOI:** 10.1038/s41598-024-60737-9

**Published:** 2024-04-30

**Authors:** Ao Wang, Shengjun Dong, Baohui Liu, Dianxiao Liu, Mingrui Zou, Yuexin Han, Lijuan Yang, Yujiu Wang

**Affiliations:** 1 Department of Cardiovascular Surgery, Binzhou Medical University Hospital, Binzhou, 256600 Shandong Province China; 2 Department of Medical Research Center, Binzhou Medical University Hospital, Binzhou, 256600 Shandong Province China

**Keywords:** Aortic dissection, Perivascular adipose tissue, Inflammation, RUNX1, Aortic diseases, Inflammation

## Abstract

The pathogenesis of aortic dissection (AD), an aortic disease associated with high mortality, involves significant vascular inflammatory infiltration. However, the precise relationship between perivascular adipose tissue (PVAT) and aortic dissection remains incompletely understood. The objective of this study is to investigate the role of PVAT inflammation in the pathogenesis of aortic dissection and identify novel therapeutic targets for this disease. The mouse model of aortic dissection was established in this study through intraperitoneal injection of Ang II and administration of BAPN in drinking water. Additionally, control groups were established at different time points including the 2-week group, 3-week group, and 4-week group. qPCR and immunohistochemistry techniques were employed to detect the expression of inflammatory markers and RUNX1 in PVAT surrounding the thoracic aorta in mice. Additionally, an aortic dissection model was established using RUNX1 knockout mice, and the aforementioned indicators were assessed. The 3T3-L1 cells were induced to differentiate into mature adipocytes in vitro, followed by lentivirus transfection for the knockdown or overexpression of RUNX1. The study aimed to investigate the potential cell-to-cell interactions by co-culturing 3T3-L1 cells with A7r5 or RAW264.7 cells. Subsequently, human aortic PVAT samples were obtained through clinical surgery and the aforementioned indicators were detected. In comparison to the control group, the aortic dissection model group exhibited decreased expression of MMP-2 and NF-κB in PVAT, while TNF-α and RUNX1 expression increased. Suppression of RUNX1 expression resulted in increased MMP-2 and NF-κB expression in PVAT, along with decreased TNF-α expression. Overexpression of RUNX1 upregulated the expression levels of NF-Κb, MMP-2, and TNF-α in adipocytes, whereas knockdown of RUNX1 exerted an opposite effect. Macrophages co-cultured with adipocytes overexpressing RUNX1 exhibited enhanced CD86 expression, while vascular smooth muscle cells co-cultured with these adipocytes showed reduced α-SMA expression. In human samples, there was an increase in both RUNX1 and MMP-2 expression levels, accompanied by a decrease in TNF-α and NF-Κb expression. The presence of aortic dissection is accompanied by evident inflammatory alterations in the PVAT, and this phenomenon appears to be associated with the involvement of RUNX1. It is plausible that the regulation of PVAT's inflammatory changes by RUNX1/NF-κB signaling pathway plays a role in the pathogenesis of aortic dissection.

## Introduction

Aortic dissection (AD) is a prevalent cardiovascular emergency characterized by sudden onset, complex and diverse clinical presentations, high mortality rates, and poor prognoses^1^. The primary pathological manifestations of AD include intimal tearing of the aorta, leading to blood flow infiltration into the arterial wall through the tear^2^. This results in the formation of both true and false lumens due to subsequent tearing of the intima and media layers. In recent years, research on the pathogenesis of AD has primarily focused on endothelial cells^3,4^, smooth muscle cells^5,6^, and fibroblasts^7^. However, limited research has been conducted on the correlation between extravascular peripheral adipose tissue (PVAT) and aortic dissection. However, limited research has been conducted on the correlation between perivascular adipose tissue (PVAT) and aortic dissection.

PVAT encompasses the adipose tissue surrounding major blood vessels, such as the aorta and microvasculature. It primarily consists of adipocytes, fibroblasts, and macrophages^8^. PVAT is intricately associated with the vessel wall from anatomical and developmental perspectives, playing a crucial role in maintaining vascular homeostasis and facilitating normal blood vessel function^9–11^. However, recent research has revealed that inflammatory PVAT is intricately linked to a range of vascular disorders, including atherosclerosis, hypertension, diabetes-related vascular diseases, and vascular aging^12^. Its primary role involves creating a localized inflammatory microenvironment by facilitating the infiltration of inflammatory cells into the vessel wall and inducing vascular remodeling. Consequently, this process contributes to or exacerbates local pathological changes in the vasculature^13–15^. Nonetheless, the precise underlying mechanism through which inflammatory PVAT contributes to the pathogenesis of AD remains elusive.

The RUNX family, consisting of RUNX1, RUNX2, and RUNX3, is a group of Runt-related transcription factors. As a well-established cancer biomarker, the involvement of RUNX in various cancers encompasses the regulation of cancer cell proliferation, metastasis, and angiogenesis^16^. However, recent study has shed light on the significant role played by RUNX1 in cardiovascular diseases such as cardiac remodeling and pulmonary hypertension. Consequently, it emerges as a potential therapeutic target^17,18^. Therefore, investigating the pathogenesis underlying the involvement of RUNX1 in aortic dissection holds immense significance for identifying potential therapeutic targets.

In this study, we have identified the involvement of RUNX1/NF-κb in the pathogenesis of aortic dissection through its regulation of PVAT-mediated inflammatory response. Furthermore, our findings suggest that inflamed adipose tissue plays a potential role in modulating VSMCs phenotypic transformation and macrophage polarization.

## Method

### Animal studies

The welfare of animals and the implementation of experimental procedures were conducted in accordance with internationally recognized ethical guidelines, ARRIVE guidelines, and the National Institutes of Health (NIH Publication 85-23, 1985 revision). The Ethics Committee of the Affiliated Hospital of Binzhou Medical College conducted a thorough review of all animal experiments (No. 20220128-75). The experiments were conducted in strict adherence to the applicable guidelines and regulations, as confirmed.

The wild-type male C57BL/6 mice were procured from Jinan Pengyue Experimental Animal breeding Co.Ltd (Jinan, China). RUNX1^flox/flox^ mice (Strain NO.T009662) were purchased from GemPharmatech(Nanjing, China). Adipo^cre+^ mice (Strain NO.T000166) were purchased from NRCMM(National Resource Center for Mutant Mice, Nanjing University) (Nanjing, China). Mice of both genes were based on C57BL/6 genetic background. Adipo^cre+^ mice strain was B6; FVB-Tg(Adipoq-cre)/Nju. According to the literature provided by the company, this line can express cre enzyme well in white adipocytes and brown adipocytes and has efficient cre enzyme expression activity without ectopic expression^19^. Genotyping PCR was performed according to the protocol from GemPharmatech or NRCMM.

The mice were housed in the animal facilities of Binzhou Medical University under specific pathogen-free conditions, residing in a controlled environment with a 12-h light/12-h dark cycle and maintaining temperature stability. They had unrestricted access to food and water. Angiotensin II (0.5 mg/ml, cat.HY-13948, MedChemExpress) was administered intraperitoneally at a dosage of 4.5 mg/kg/day along with BAPN (cat.HY-Y1750, MedChemexpress) in the drinking water at a concentration of 0.1 mg/kg/day for a duration of 4 weeks to establish a mouse model of aortic dissection. The wild-type male mice, aged 4–6 weeks, were randomly assigned to the control group, 2-week group, 3-week group, and 4-week group. The modeling for the 2-week group, 3-week group, and 4-week group commenced in the third week, second week, and first week respectively. The control group received only intraperitoneal injection of normal saline and access to drinking water without any drugs. Male RUNX1 flox/flox cre + mice aged 4–6 weeks were selected, and the model was established using the aforementioned method for a duration of 4 weeks. At the conclusion of the fourth week, all surviving mice in each group were anesthetized with 1.25% bromoethanol (REF.M2910, Nanjing Aibei Biotechnology Co. Ltd.) and euthanized by cervical dislocation, after which the aorta was promptly dissected. The thoracic aorta's partial PVAT and thoracic aorta with PVAT were meticulously excised. PVAT was immediately preserved in liquid nitrogen while the thoracic aortas with PVAT were stored in paraformaldehyde.

### Histology and immunohistochemistry

The thoracic aorta, fixed in paraformaldehyde, was embedded in paraffin and sectioned into 4 μm thick slices. The sections were subsequently stained with hematoxylin and eosin (H&E) as well as Elastica van Gieson (EVG). Additionally, immunohistochemistry was performed using anti-RUNX1 (1:200; cat. GB11797; servicebio), anti-MMP2 (1:1000; cat. GB11130; servicebio), anti-TNF-α (1:200; cat. GB11188; servicebio), anti-F4/80 (1:500; cat. GB113373; servicebio) antibodies, and the Universal two-step detection kit (Beijing Zhongshan Jinqiao Biotechnology Co.Ltd) following the manufacturer's instructions. The immunohistochemical results were analyzed using Image J software to determine the relative absorbance.

### Cell culture

The RAW264.7, 3T3-L1, and A7R5 cell lines along with their respective complete media were procured from Procell Life Science & Technology Co., Ltd.

The overexpression lentivirus targeting RUNX1 was procured from Vigenebio Technologies (Shandong, China), while the shRNA lentivirus targeting RUNX1 was obtained from GeneChem (Shanghai, China). The 3T3-L1 cells were genetically modified to either overexpress or knockdown RUNX1 using lentiviral transfection technology in accordance with the manufacturer's instructions. According to the results of the preliminary experiment, the MOI value of 3T3-L1 cells was 100 (Fig. S1). The infection system was calculated by the formula (MOI = virus titer (TU/ml) × virus volume (ml)/cell number) and virus titer. A 100 μl infection system was established in 1.5 ml EP tubes. The mixture was gently blown with a pipettor and placed in a cell incubator at 37 ℃for 0.5-1 h. The whole infected system was transferred to a 6-well plate pre-supplemented with 2 ml of complete medium by pipettor and incubated at 37 °C in a 5% CO^2^ incubator. After 12 h of culture, the medium containing virus was replaced with fresh complete medium. The expression of GFP green fluorescence was observed under a fluorescence microscope 72 h after transfection, and puromycin was added to screen the stable virus strain, and the screening period was 2–10 days. After the cells grew stably, the expression of RUNX1 was detected by Western blot, and the subsequent experiments were performed.

The induced 3T3-L1 cells and RUNX1 knockdown 3T3-L1 cells were treated with lipopolysaccharide (LPS) at a concentration of 1 μg/ml (cat. L8880, Solarbio) for 24 h to induce an inflammatory phenotype.

The co-cultures were conducted using a co-culture chamber system (Corning, NY) for a duration of 2 days. The vector group, 3T3-L1 cells overexpressing RUNX1, and 3T3-L1 cells with RUNX1 knockdown were seeded in the upper chamber, while A7R5 and RAW264.7 cells were seeded in the lower chamber. After co-culture, the lower chamber cells were collected for subsequent experiments. The coculture system was operated in detail as follows.

3T3-L1 cells, RAW264.7 cells and A7r5 cells were suspended and counted. Then 2 ml 3T3-L1 cell complete medium was added to the Transwell chamber and the lower layer of the Transwell chamber. 3T3-L1 cells were seeded at 1 × 10^5^/ml in Transwell chambers and manipulated according to the induced differentiation procedure. At 10-Day, 2 ml of complete medium (15% fetal bovine serum) was added to each well of sterile 6-well plates, and RAW264.7 and A7r5 cells were inoculated at 1 × 10^5^/ml, respectively. New medium was replaced every 48 h. When the induction of 3T3-L1 cells was complete and the density of RAW264.7 cells or A7r5 cells was 70–80%, the Transwell chamber for culturing 3T3-L1 cells was transferred to the 6-well plate for culturing RAW264.7 cells or A7r5 cells. Complete medium (15%FBS) was used for the Transwell chamber and the lower layer of the chamber. The co-culture system was incubated in a 5% CO^2^ incubator at 37 °C for 24 h to complete the culture.

### 3T3-l1 cells were induced to differentiate into adipocytes

A differentiation solution was prepared by combining 3T3-L1 cell complete medium (100 ml), insulin solution (1 mg/ml, PB180432, Procell) (1 ml), IBMX (50 mm, HY-12318, MedChemExpress) (1 ml), dexamethasone solution (10 mm, HY-14648, MedChemExpress) (100μL), and rosiglitazone solution (10 mM, cat.HY-17386, MedChemExpress) (20μL). The differentiation medium B consists of 100 ml of 3T3-L1 complete medium and 1 ml of insulin solution (1 mg/ml). The mixture was thoroughly mixed and then filtered to remove bacteria before being stored at 4 °C. The 3T3-L1 cells were cultured until reaching complete confluence using a complete medium, which occurred on Day-2. On Day 0, differentiation medium A was utilized for the culture, with medium exchanges performed once every 2 days. The cells were cultured with differentiation medium B from Day 6 until the completion of Day 8 induction, and the medium was changed every two days.

### Western blot

The collected cells were lysed using a mixture of RIPA lysate (Beyotime Biotechnology, China) and protease inhibitor (Beyotime Biotechnology, China) at a ratio of 100:1. The primary antibodies used for incubation were anti-RUNX1/2/3 (1:500; cat.BM3876; Boster), anti-GAPDH (1:1000; cat.BM4265; Boster), and anti-α-SMA (1:1000; cat.#14968S; Cell Signaling) to ensure comprehensive coverage.

### Quantitative reverse transcription-polymerase chain reaction

The extraction of total RNA from cells and tissues was performed using the Eastep® Super Total RNA Extraction Kit (Promega, USA) following the manufacturer's protocol. The total RNA was extracted and subjected to reverse transcription using the RevertAid RT Kit (cat. K1691, Thermo) according to the manufacturer's protocol. The primers were synthesized by Sangon (Shanghai, China) according to the provided sequences listed in Table S1.

### Human studies

This study adhered to the principles delineated in the Declaration of Helsinki and obtained approval from the Ethics Committee of Binzhou Medical College (KYLL-2022-77). The experiments were conducted in strict adherence to the applicable guidelines and regulations, as confirmed. Informed consent was obtained from patients and their families, who also signed the informed consent form.

The human aortic PVAT was collected intraoperatively from the Department of Cardiovascular Surgery at the Affiliated Hospital of Binzhou Medical University. In patients with aortic dissection, PVAT was located in the ascending aorta or aortic arch adjacent to the proximal tear of the aortic dissection. Without any form of electrical stimulation or other external stimuli, PVAT was carefully dissected using tissue scissors. In the control group, aortic PVAT was obtained from the root of the aortic artery during other surgical procedures without aortic dissection, such as coronary artery bypass grafting and valve-related surgery. The collection of PVAT did not result in any additional harm to the patients, and all harvested tissues were planned for resection due to underlying diseases. A total of 75 patient samples were included in this study, including 35 patients with aortic dissection and 40 patients without aortic dissection. The inclusion and exclusion criteria for clinical patient sample acquisition are shown in Table S2.

### Statistical analysis

Using GraphPad Prism 9 software to deal with the experimental data analysis, the results are conducted through $\overline{x}$ ± s said. Firstly, the normality and homogeneity of variance of the experimental data were analyzed. When comparing the differences between the two groups, t test was used if the distribution was normal, and M–W *U* test was used if the distribution was not normal. When comparing the differences between multiple groups, one-way ANOVA test was used if the distribution was normal and the variance was homogeneous, Welch ANOVA test was used if the distribution was normal and the variance was uneven, and Kraskal Wall's test was used if the distribution was not normal. The test results showed that P < 0.05, indicating that there was a statistically significant difference between the groups.

## Result

### PVAT of AD mice exhibits inflammatory changes

**Figure 1:**
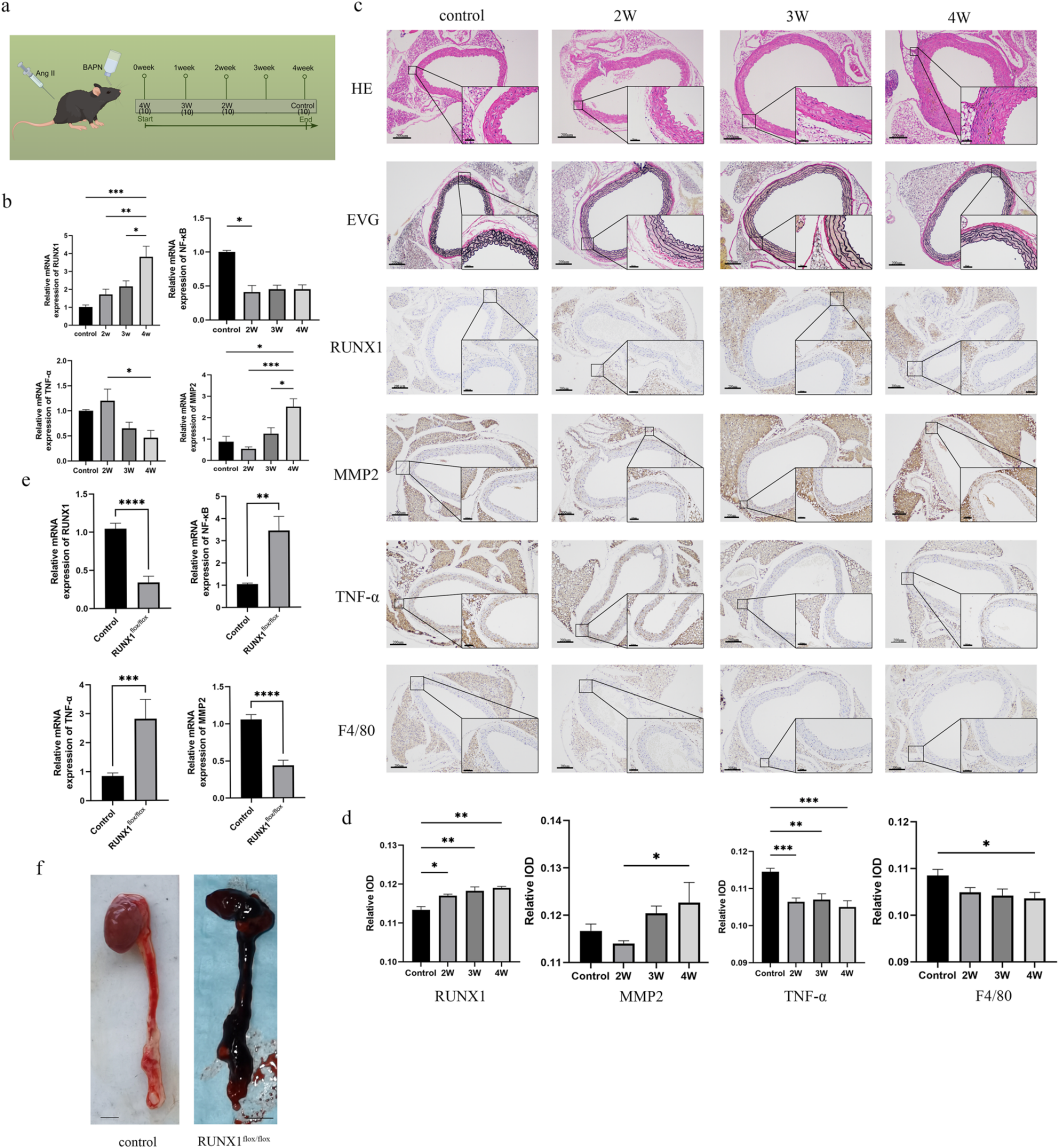
Analysis of in vivo results. (**a**) Schematic diagram depicting the establishment of an animal model for aortic dissection In vivo experiments were divided into 4 groups with 10 mice in each group. (**b**) Analysis of qPCR results for RUNX1, NF-κB, MMP-2, and TNF-α in thoracic aortic PVAT of wild-type mice with aortic dissection. (**c**, **d**) Analysis of IHC results for RUNX1, F4/80, MMP-2, and TNF-α in thoracic aortic PVAT of wild-type mice with aortic dissection. (**e**) qPCR analysis of RUNX1, NF-κB, MMP-2, and TNF-α in the thoracic aortic PVAT of RUNX1^flox/flox^ mice with aortic dissection. (**f**) Results of aorta gross specimens from AD RUNX1^flox/flox^ mice and control mice. When comparing the differences between the two groups, t test was used for analysis if the distribution was normal. When comparing the differences between multiple groups, one-way ANOVA test was used if the distribution was normal and the variance was homogeneous. Data are shown as $\overline{x}$ ± s. *P < 0.05, **P < 0.01, ***P < 0.001, ****P < 0.0001.

The mouse model of aortic dissection was established through intraperitoneal injection of AngII and BAPN in the drinking water, enabling the detection of inflammatory factors and RUNX1 expression in the aortic PVAT at different stages of aortic dissection. (Fig. 1a) The results demonstrated divergent trends in the expression of TNF-α and MMP-2. Specifically, TNF-α exhibited a significant decrease at the 4th week, whereas MMP-2 displayed an opposing pattern. Furthermore, the expression of NF-κb, a pivotal signaling pathway associated with inflammation, experienced a notable reduction during the second week and subsequently maintained relative stability in the subsequent weeks (Fig. 1b). The histological staining revealed that as the modeling time increased, there was a progressive thickening of the wall accompanied by disorganization and disruption of elastic fibers and collagen (Fig. 1c). The IHC results revealed a significant increase in MMP-2 expression in PVAT at week 4, whereas TNF-α exhibited a significant decrease at week 2 (Fig. 1c,d). Furthermore, we conducted an analysis on the macrophage marker F4/80 and observed a significant reduction in its expression at week 4 compared to the control group (Fig. 1c,d).

### The inflammatory changes in PVAT are associated with RUNX1

In the thoracic aortic PVAT of mice with AD, we observed a significant upregulation in the expression of RUNX1 at week 4 (Fig. 1b). The IHC results revealed a significant upregulation of RUNX1 expression during the second, third, and fourth weeks in comparison to the control group (Fig. 1c,d). The role of RUNX1 in the PVAT of aortic dissection was further investigated by generating Runx1-specific fat knockout AD mice using the RUNX1^flox/flox^ mouse strain. Compared to the control group, the RUNX1^flox/flox^ group exhibited a significant reduction in MMP2 expression and a notable elevation in NF-κb and TNF-α expression within the PVAT at 4 weeks post establishment of AD (Fig. 1e). The severity of aortic dissection was significantly increased in the RUNX1^flox/flox^ group (Fig. 1f).

### RUNX1 is implicated in adipocyte inflammation

**Figure 2:**
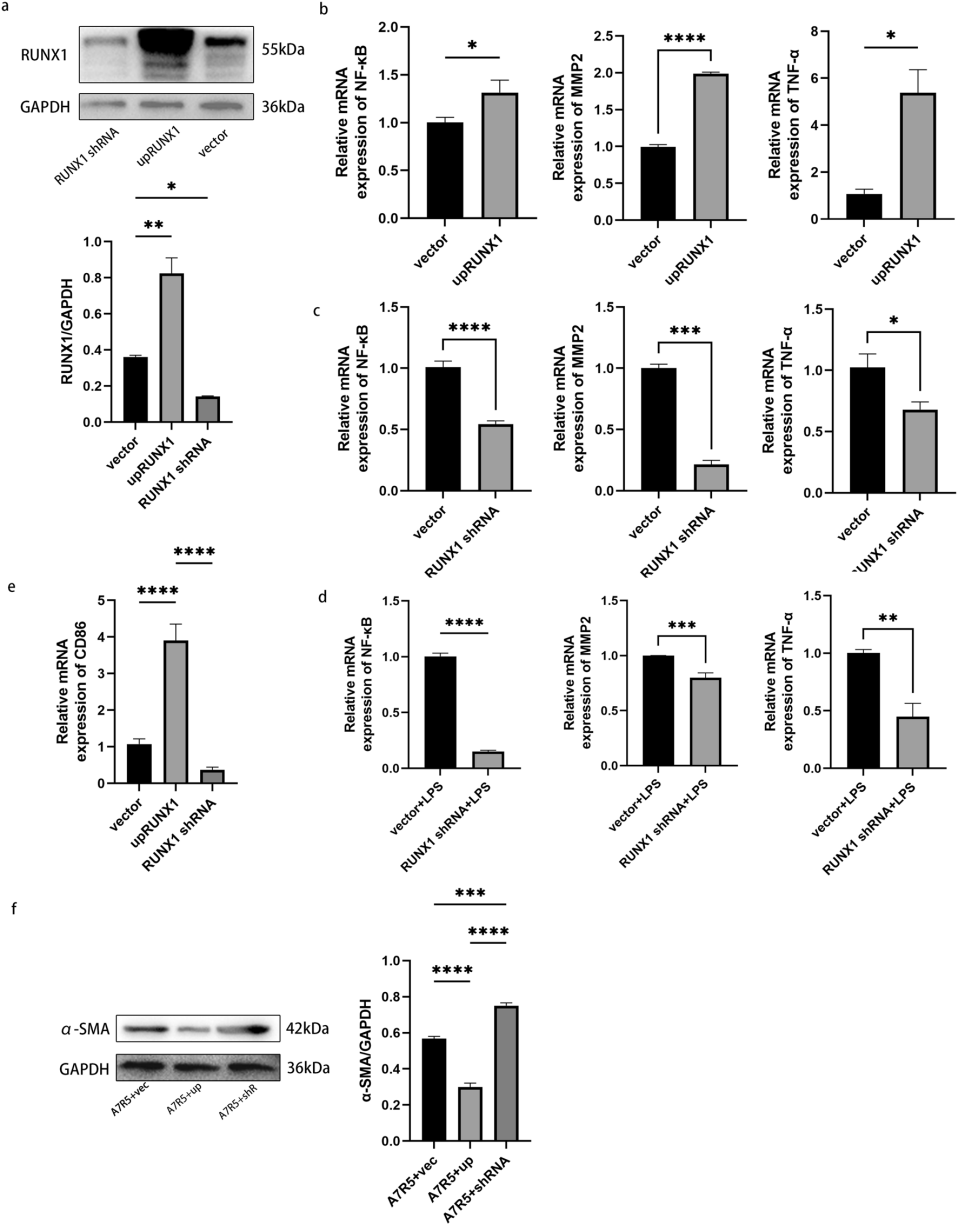
(**a**) The construction of 3T3-L1 cells with either knockdown or overexpression of RUNX1 was performed to validate the findings. (**b**) Results of NF-κB, MMP-2, and TNF-α qPCR expression in 3T3-L1 cells overexpressing RUNX1 stable strains. (**c**) NF-κB, MMP-2 and TNF-α qPCR expression results of RUNX1 stable knockdown strain in 3T3-L1 cells. (**d**) qPCR results of NF-κB, MMP-2 and TNF-α expression in 3T3-L1 cells with knockdown RUNX1 stable strain treated with LPS for 24 h. (**e**) CD86 qPCR results of RAW264.7 after co-culture with 3T3-L1 cells with RUNX1 overexpression or knockdown. (**f**) Results of α-SMA after co-culture of A7r5 with RUNX1 overexpression or knockdown in 3T3-L1 cells. All in vitro experiments in this figure were performed with three technical replicates and six biological replicates. When comparing the differences between the two groups, t test was used for analysis if the distribution was normal. When comparing the differences between multiple groups, one-way ANOVA test was used if the distribution was normal and the variance was homogeneous. Data are shown as $\overline{x}$ ± s. *P < 0.05, **P < 0.01, ***P < 0.001, ****P < 0.0001.

We investigated the underlying mechanism of RUNX1 in adipocyte inflammation by inducing differentiation of 3T3-L1 cells into mature adipocytes. Stable cell lines with either overexpression or knockdown of RUNX1 were generated through lentiviral transfection of 3T3-L1 cells (Fig. 2a). The levels of NF-κB, MMP-2, and TNF-α were significantly upregulated in the group with RUNX1 overexpression (Fig. 2b). The expression of NF-κB, MMP-2, and TNF-α was significantly reduced in the RUNX1 knockdown group (Fig. 2c). This effect remained evident even after 24 h of LPS treatment (Fig. 2d). The effect of RUNX1 overexpression or knockdown in adipocytes on macrophages or vascular smooth muscle cells was investigated by establishing a non-contact co-culture system to examine cell-to-cell interactions. The results demonstrated a significant increase in the M1 macrophage marker CD86 expression in RAW264.7 cells upon RUNX1 overexpression, compared to both the Vector group and the RUNX1 knockdown group. However, there was no significant difference observed between the RUNX1 knockdown group and the Vector group (Fig. 2e). The expression of α-SMA, a phenotypic marker for contraction in A7r5 cells, was significantly reduced in the group overexpressing RUNX1 and increased in the group with knockdown of RUNX1 (Fig. 2f).

### RUNX1 changes can be detected in the PVAT of AD human

**Figure 3:**
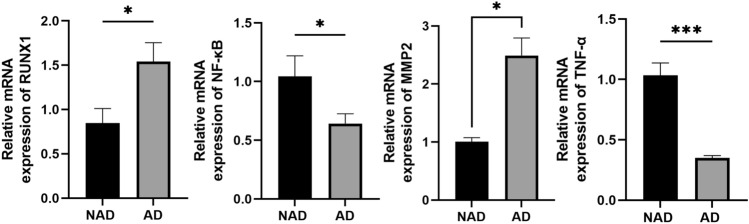
qPCR analysis of RUNX1, NF-κB, MMP-2 and TNF-α in aortic PVAT in patients with aortic dissection compared with control group. When comparing the differences between the two groups, t test was used for analysis if the distribution was normal. Data are shown as $\overline{x}$ ± s. *P < 0.05, **P < 0.01, ***P < 0.001, ****P < 0.0001.

In this study, the expression of RUNX1 and inflammatory factors was also assessed in human PVAT. Consistent with the findings from animal experiments, there was a significant upregulation of RUNX1 and MMP-2 expressions, while NF-κB and TNF-α expressions were significantly reduced in PVAT surrounding the thoracic aorta of patients with AD (Fig. 3).

## Discusstion

The destruction of elastic fibers and infiltration of inflammatory cells in the aortic wall are key pathological features of aortic dissection. Local inflammation exacerbates the development of AD^20^. Perivascular adipose tissue, which is in close proximity to the vessel wall, plays a crucial role in regulating vascular homeostasis by modulating vascular function through paracrine and other signaling pathways^21,22^. The pro-inflammatory phenotype of PVAT plays a crucial role in the development of vascular wall inflammation, which can contribute to various vascular diseases such as atherosclerosis^22,23^. The precise contribution of PVAT in the pathogenesis of aortic dissection remains incompletely understood.

The present study revealed evident inflammatory alterations in thoracic aortic PVAT during the progression of aortic dissection, characterized by reduced expression of the pro-inflammatory cytokine TNF-α and enhanced expression of MMP-2. These changes were concomitant with increased expression of RUNX1 and decreased expression of the inflammation-related NF-κB pathway. Notably, knockout of RUNX1 resulted in contrasting outcomes for the expressions of TNF-α, MMP-2, and NF-κB. The findings suggest that the inflammatory response mediated by aortic PVAT plays a significant role in the pathogenesis of aortic dissection, and this alteration is closely associated with RUNX1. The transcription factor RUNX1 exerts diverse functions in various diseases, particularly exerting an impact on inflammation^17^. The study conducted by Kubin et al. revealed a significant association between up-regulation of RUNX1 and the presence of cardiomyocyte inflammation, as well as an increased infiltration of myocardial macrophages in mice^24^. The inhibition of toll-like receptor 4 by RUNX1 in acute lung injury serves to suppress inflammation in lung epithelial cells^25^. The expression of the macrophage marker F4/80 in PVAT was notably reduced during the progression of aortic dissection, potentially indicating infiltration of macrophages into the vessel wall and subsequent promotion of inflammatory processes. The adventitia is known to be infiltrated by macrophages prior to the intima and media, facilitating their entry into the vessel wall and subsequent involvement in vascular remodeling^26,27^.

In vitro experiments demonstrated that upregulation of RUNX1 expression in mature adipocytes significantly increased the expression levels of MMP-2, TNF-α, and NF-κB. Conversely, knockdown of RUNX1 expression resulted in a decrease in these aforementioned indicators. It is worth noting that the expression trends of TNF-α and NF-κB in vitro experiments were not consistent with those in vivo experiments. In view of the discordant results, we made two hypotheses. First, as a transcription factor regulating a variety of physiological processes, RUNX1 has different and even opposite roles in different tissues and cells. Previous studies have shown that RUNX1 inhibits NF-κB signaling pathway by inhibiting IκB kinase β in lung epithelial cells, thereby reducing LPS-induced lung inflammation^28^. Li et al. showed that 6-gingerol significantly inhibited LPS-induced activation of NF-κB signaling pathway in type II alveolar epithelial cells by directly binding to RUNX1 and inhibiting its activity, thus reducing lung injury and pulmonary edema^29^. However, RUNX1 interacts with NF-κB subunit p50 in macrophages, thereby enhancing NF-κB activity and promoting signaling pathway activation, increasing the production of inflammatory factors such as IL-1β and IL-6^30^. It has been shown that RUNX1 promotes the production of proinflammatory factors such as TNF-α by activating TLR1/2 and TLR4 signaling in neutrophils^25^. In conclusion, RUNX1 has different effects on inflammatory pathways such as NF-κB signaling pathway in different tissue cells. In this study, the tissue obtained in the in vivo experiment was the PVAT of the thoracic aorta, which contained many cell types such as adipocytes, macrophages, fibroblasts and so on. The expression of related indicators of certain cell types was not detected by single-cell sequencing technology during the study, while the in vitro experiment used a single type of cell sample. Therefore, the results of some indicators in vivo and in vitro experiments were inconsistent. Second, this study generated a mature adipocyte model by inducing 3T3-L1 cells, which have the potential to switch to a macrophage-like phenotype. Previous studies have shown that MiR-27a intervention can promote the transformation of 3T3-L1 cells into macrophage-like characteristics, enhance their phagocytosis and migration function, and increase the expression of TNF-α, MCP-1, IL-1β and other pro-inflammatory factors^31^. Recent studies have shown that MiR-27a mediates increased glucose uptake and decreased insulin resistance in 3T3-L1 adipocytes by regulating the PPAR-γ-PI3K /AKT-GLUT4 signaling axis^32^. Xiao et al. showed that RUNX1 knockdown in 3T3-L1 adipocytes promoted their adipogenic differentiation, while RUNX1 overexpression significantly reduced the expression of adipocyte marker genes PPARγ, C/EBPα and FABP4 and inhibited cell adipogenic differentiation^33^. Therefore, in this study, 3T3-L1 adipocyte model was used to study the role of RUNX1 in adipocyte inflammation. During the study, 3T3-L1 adipocytes may have potential macrophage-related characteristics transformation, which leads to inconsistent expression of some inflammatory indicators with the results of relevant indicators in vivo experiments. However, the above two hypotheses have not been proved in this study and cannot fully explain the contradictory results. Based on the results of the present study alone, it can be demonstrated that RUNX1 has an effect on the inflammatory response of adipocytes.

Adipocytes overexpressing RUNX1 facilitated the polarization of macrophages towards the M1 phenotype and similarly promoted the loss of contractile phenotype in vascular smooth muscle cells. On the other hand, adipocytes with reduced RUNX1 expression did not exhibit such functionality. The data suggest that RUNX1 in adipocytes not only promotes heightened inflammation within adipocytes, but also enhances cellular capacity to induce macrophage polarization and phenotypic alterations in smooth muscle cells. The results of this study showed that RUNX1 overexpression significantly enhanced the inflammatory response and increased the expression of TNF-α and MMP-2 in 3T3-L1 adipocytes. As a powerful proinflammatory factor, TNF-α can not only promote the polarization of macrophages to M1 macrophages by activating macrophages, but also induce apoptosis or phenotypic transformation of smooth muscle cells^34^. MMP-2 is highly expressed in a variety of cardiovascular diseases such as myocardial infarction and hypertensive heart disease. Mmp-2 can not only activate macrophages and stimulate systemic inflammation, but also participate in tissue remodeling by promoting the phenotypic transformation of smooth muscle cells^35,36^. Lian et al.^20^ discovered that the metabolic reprogramming of macrophages triggers HIF-1α activation, which in turn promotes aortic dissection and leads to an increased polarization of macrophages towards a proinflammatory M1 phenotype during the disease progression. The presence of vascular smooth muscle cells is crucial for maintaining the stability and functionality of the vascular structure, while alterations in their quantity and phenotype serve as significant mechanisms underlying aortic dissection^37^. In this study, the presence of inflammation and elevated levels of RUNX1 were also observed in the PVAT of individuals with aortic dissection, indicating that RUNX1 may contribute to disease pathogenesis through PVAT-mediated inflammation.

The results of this study indicate that RUNX1 plays an important role in the pathogenesis of aortic dissection and is a potential target for disease prevention and testing. Through clinical transformation, in the future, a variety of strategies such as small molecule inhibitors, monoclonal antibodies, gene editing technology or RNA interference may be used to target RUNX1 in the aortic PVAT for the prevention of aortic dissection. At present, in the study of acute myeloid leukemia, it has been shown that the survival rate of AML mouse models with mtRUNX1 expression can be effectively improved by CRISPR/Cas9 editing technology and the application of RUNX1 inhibitors^38,39^. In addition, there is no specific test index for aortic dissection at present, and RUNX1 may become a new test index for aortic dissection in the next research.

This study still has limitations, mainly reflected in the following aspects. First, although RUNX1 was knocked out in the aortic PVAT in mice used for in vivo experiments, RUNX1 was knocked out in adipose specific mice, and RUNX1 was also knocked out in fat in other parts of the body, so there may be additional effects on experimental animals and bias the results. The longest time of aortic dissection animal model used in this study was 4 weeks. Considering the nature of chronic pathological changes of aortic dissection, 4 weeks of observation time may not fully explain the long-term development of the disease. Second, because 3T3-L1 cells have the potential for macrophage-related alterations, which were not further explored in this study, there may be potential additional phenotypic alterations in the effects of RUNX1 on adipocytes. Thirdly, in the clinical sample detection experiment, samples from non-aortic dissection patients were used as the control group in this study. Because it is extremely difficult to obtain aortic samples from healthy people, the experimental results may be biased. These limitations should be avoided by further seeking to establish more accurate animal and cell models in future studies.

In conclusion, the present study has identified the involvement of RUNX1/NF-κB in aortic dissection development through its regulation of PVAT inflammatory response. This suggests that targeting PVAT inflammatory response and RUNX1 could be promising strategies for treating aortic dissection.

### Supplementary Information

Supplementary Figure S1.

Supplementary Figure S2.

Supplementary Table S1.

Supplementary Table S2.

## Data Availability

The datasets used and/or analysed during the current study available from the corresponding author on reasonable request.
