# Supplementary figures and images for: The role of RUNX1/NF-κB in regulating PVAT inflammation in aortic dissection
Source: Sci Rep. 2024 Apr 30;14:9960. doi: 10.1038/s41598-024-60737-9 (PMC11063189; doi:10.1038/s41598-024-60737-9)

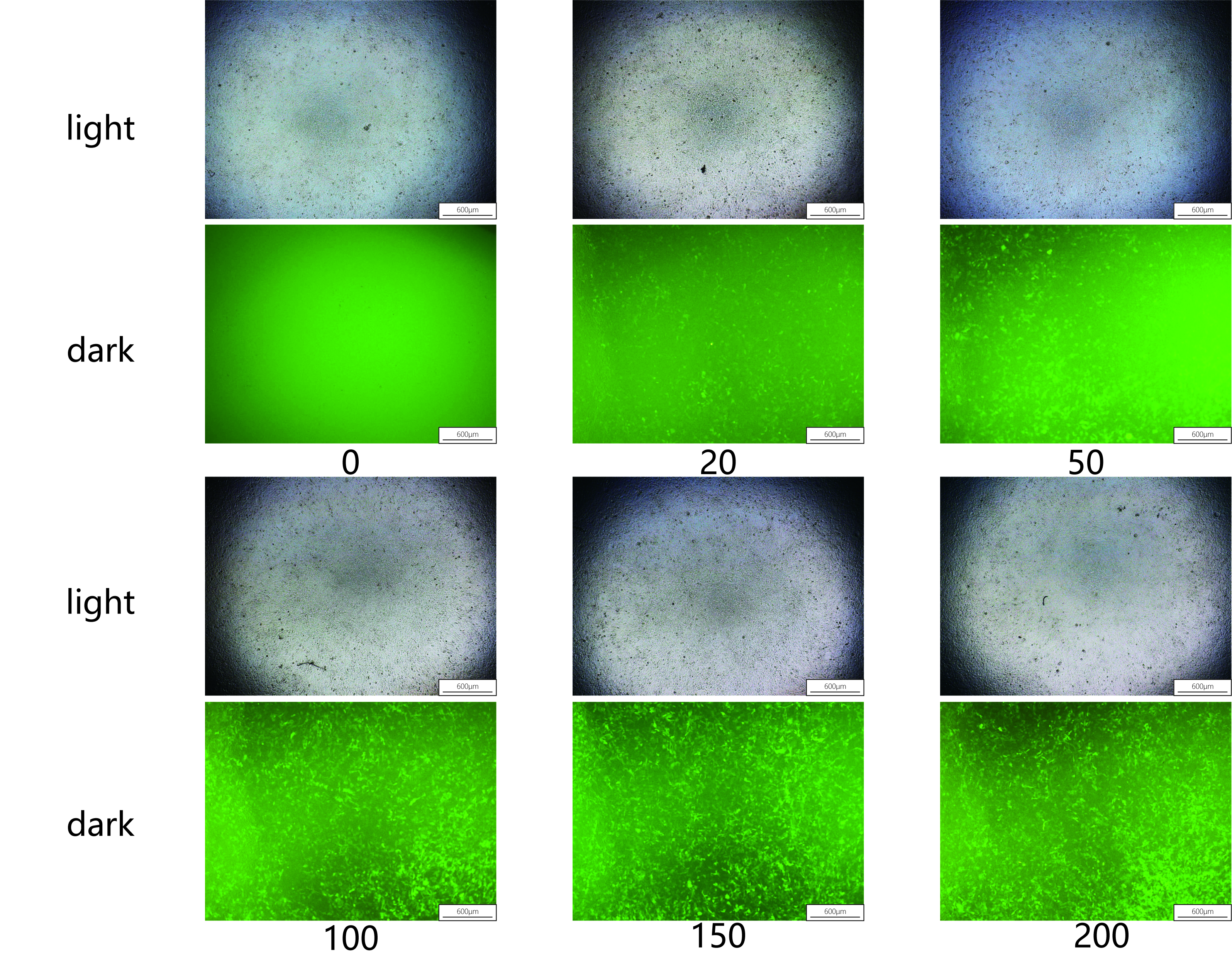

Supplement: Supplementary file 1 — Supplementary Figure S1. [file 41598_2024_60737_MOESM1_ESM.tif]

Figure2a

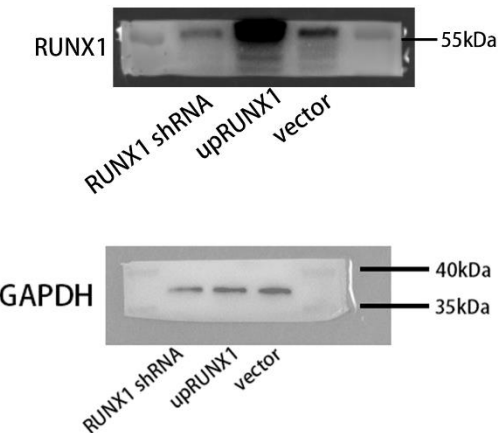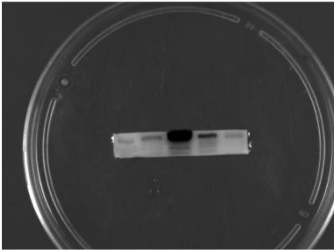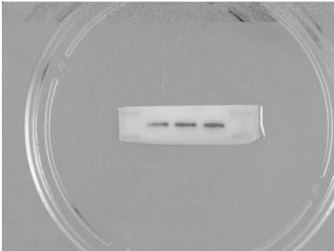

Figure2b

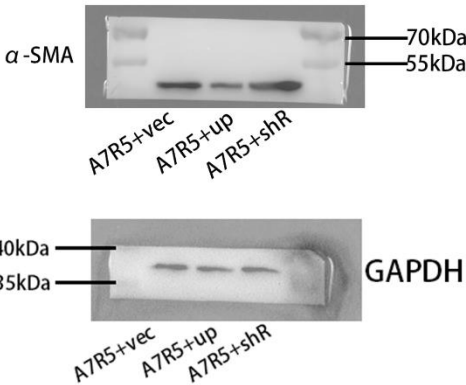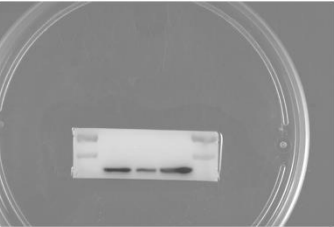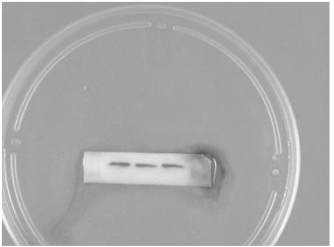

Supplement: Supplementary file 2 — Supplementary Figure S2. [file 41598_2024_60737_MOESM2_ESM.pdf]
