# Supplementary material for: The role of RUNX1/NF-κB in regulating PVAT inflammation in aortic dissection
Source: Sci Rep. 2024 Apr 30;14:9960. doi: 10.1038/s41598-024-60737-9 (PMC11063189; doi:10.1038/s41598-024-60737-9)
Supplement: Supplementary file 4 — Supplementary Table S2. [file 41598_2024_60737_MOESM4_ESM.docx]

Inclusion and exclusion criteria

1. Inclusion criteria

Inclusion criteria for the study were as follows:

① Patients of both sexes, older than 30 years old;

② A definite diagnosis of aortic dissection or non-aortic disease was made during operation;

③ patients with onset of aortic dissection less than 48 hours;

④ The patient had no history of thoracotomy;

⑤ patients whose samples passed the quality inspection were obtained.

1. Exclusion Criteria

Exclusion criteria for this study were as follows:

① patients who had undergone previous thoracotomy or aortic intervention;

② patients who could not obtain samples due to critical condition during the operation;

③ patients with multiple aortic plaques or severe calcification that may cause additional harm to obtain samples;

④ patients with insufficient aortic surface fat to obtain adequate samples;

⑤ patients whose samples failed the quality inspection were obtained.
